# A review on factors related to patient comfort experience in hospitals

**DOI:** 10.1186/s41043-023-00465-4

**Published:** 2023-11-08

**Authors:** Yu Tian

**Affiliations:** Department of Mechanical Engineering, Faculty of Engineering, Universiti Malaya, 50603 Kuala Lumpur, Malaysia

**Keywords:** Physical environment, Thermal comfort, Indoor air quality, Air purification, Hospital environment

## Abstract

The creation of a welcoming hospital atmosphere is necessary to improve patient wellbeing and encourage healing. The goal of this study was to examine the variables affecting hospitalised patients' comfort. The study procedure included a thorough search of the Web of Science and Scopus databases, as well as the use of software analytic tools to graphically map enormous literature data, providing a deeper understanding of the linkages within the literature and its changing patterns. Insights from a range of disciplines, including engineering, psychology, immunology, microbiology, and environmental science, were included into our study using content analysis and clustering approaches. The physical environment and the social environment are two crucial factors that are related to patient comfort. The study stress the need of giving patient comfort a top priority as they heal, especially by tackling indoor air pollution. Our research also emphasises how important hospital care and food guidelines are for improving patient comfort. Prioritising patients who need specialised care and attention, especially those who have suffered trauma, should be the focus of future study. Future research in important fields including trauma, communication, hospital architecture, and nursing will be built on the findings of this study. To enhance research in these crucial areas, worldwide collaboration between experts from other nations is also advised. Although many studies stress the significance of patient comfort, few have drawn conclusions from a variety of disciplines, including medicine, engineering, immunology, microbiology, and environmental science, the most crucial issue of thoroughly researching the improvement of patient comfort has not been addressed. Healthcare workers, engineers, and other professions will benefit greatly from this study's investigation of the connection between hospital indoor environments and patient comfort.

## Introduction

Patient comfort refers to the sense of happiness, relaxation, and satisfaction that patients experience throughout their medical care. It is of utmost importance in healthcare because it greatly influences patients' well-being and their perception of the overall process, leading to faster recovery and improved health outcomes. Indoor air quality (IAQ), airflow, and ventilation systems are factors that significantly impact the physical environment of hospitals, thus affecting patient comfort. Additionally, the social and humanistic environment of the hospital is greatly influenced by factors such as nursing and diet, privacy, and communication. By creating a relaxed and pleasant environment, it is possible to reduce worry and anxiety and provide a positive and comfortable experience. In the era following the pandemic, there is a heightened focus on air quality, making it highly significant to investigate the influence of IAQ on patient comfort. Optimal air quality plays a pivotal role in establishing a soothing and curative atmosphere for patients, mitigating the risk of infections, and enhancing the well-being of patients [1]. People spend 90% of their time in a built environment [2], and around 87% of their time is spent indoors [3]. Elderlies who have moderate to severe Chronic Obstructive Pulmonary Disease (COPD) and lung function impairment, in particular, go outside less frequently (46%), and spend just around 2 h outside on average [4]. Poor IAQ can lead to discomfort, irritation, and respiratory system problems. Furthermore, many countries have regulations and guidelines regarding IAQ in medical facilities [5, 6]. Improving IAQ can also help hospitals become more energy-efficient [7], save costs, and improve patient comfort and health [8]. Achieving energy reductions of up to 30% is feasible by ensuring optimum thermal comfort and using HVAC systems for air conditioning [9]. Respecting patients' privacy and ensuring adequate personal space is crucial for their comfort. Clear and empathetic communication from healthcare professionals, including transparent explanations of medical procedures, diagnoses, and treatment options, helps patients feel more at ease and in control of their healthcare journey [10]. Encouraging positive social interactions among patients and with healthcare staff can create a sense of community and reduce feelings of isolation [11]. Besides, providing high-quality care can meet the patient's emotional and psychological needs, which need to focus on patients' comfort experience and take measures to address any concerns or issues [12]. Furthermore, appropriate seating, clean restrooms, and nourishing meals are essential for patient comfort. Ensuring these fundamental needs helps patients feel cared for and promotes their general well-being. By analyzing relevant literature, the study will identify key factors that contribute to or hinder patient comfort. The findings will provide insights into different dimensions of patient comfort, guiding healthcare providers and policymakers in making informed decisions and implementing strategies to enhance comfort.

The study contributes substantially to the work of healthcare professionals, engineers, and psychologists. (1) For hospitals, improving the efficacy of medical interventions and patients' overall quality of life promotes good communication and trust between patients and medical staff, leading to better medical services and treatment effects [13–15]. (2) In terms of air quality, poor IAQ can lead to airborne diseases and infections, especially in medical environments where patients have weak immune system resistance [16]. Indoor air pollutants can trigger allergic diseases and affect personal health, even increasing mortality rates. Research has found a correlation between airborne particle pollution and death rates. Adult mortality rates will increase by 0.8–3% daily for every 10 μg m^−3^ increase in PM_10_ [17]. (3) Regarding patient comfort, studies have shown that a vast majority of critically ill patients in intensive care units (ICUs), ranging between 55 and 75%, experience moderate to severe pain [18]. Addressing the comfort needs of patients can help reduce patients' anxiety and stress, promoting faster recovery for patients [12]. Additionally, a relaxed atmosphere in hospitals helps patients feel at ease, reducing anxiety and stress, preventing physiological reactions, and meeting the requirements and expectations of patient families and hospital visitors, ultimately promoting patient comfort [19]. Despite the fact that patient comfort has been emphasised repeatedly in studies, the most important issues surrounding patient comfort improvement and its major determinants have not been thoroughly investigated across a variety of disciplines. This study emphasises that improving patient comfort, especially for trauma patients, may be accomplished by giving attention to indoor air pollutants in patient health recovery. These results offer insightful recommendations for enhancing medical services and enhancing the patient experience as a whole. These findings will serve as the foundation for further study in crucial sectors including trauma, communication, hospital architecture, and nursing. In summary, by addressing the elements that contribute to a satisfying healthcare experience, healthcare providers can create an environment that promotes patient well-being, satisfaction, and better health outcomes.

## Methodology

**Fig. 1:**
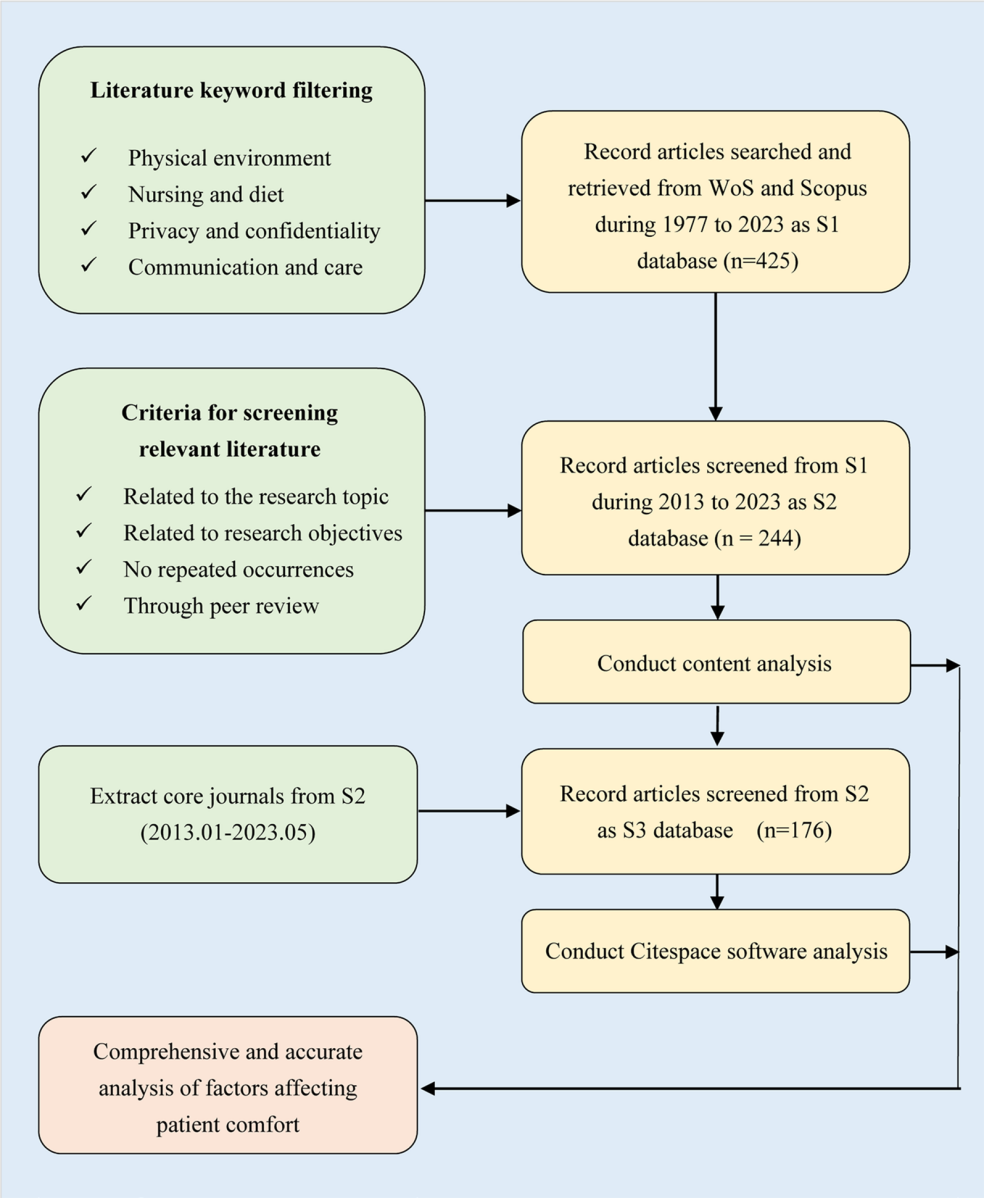
Systematic approach in retrieving articles for review purposes

In this literature review, the methods used include content analysis and CiteSpace software analysis. The software name for drawing Figs. 3, 6, 7, and 8 is CiteSpace. The process of content analysis involved defining objectives and scope, collecting, screening, and evaluating literature, extracting and organizing information, analyzing and synthesizing findings, and identifying factors influencing the patient comfort experience. Figure 1 shows the specific research process. This study uses the PICO framework to thoroughly search for pertinent papers for this investigation, which involves the classification of keywords based on population, intervention, comparison, and outcome. Specifically, the search focused on the following categories:

- Population: Patients—The study targeted articles that involved or pertained to individuals receiving healthcare services, excluding hospital visitors.
- Intervention: Not applicable—The search did not prioritize papers based on a specific intervention or treatment method.
- Comparison: Factors, components, impacts—The study aimed to explore articles that discussed various factors, components, or effects related to the research topic.
- Outcome: Comfort, satisfaction- The search was to identify papers that reported findings related to patient comfort or other relevant things.

By utilizing the PICO framework, this study aimed to gather relevant literature that addresses the specific aspects of the research question, facilitating a comprehensive analysis and potentially contributing valuable insights to the field. In Fig. 1, the study searched for keywords in the Web of Science (WoS) and SCOPUS databases, creating the literature dataset S1, which refers to the articles relevant to the study from 1977 to 2023. To obtain the literature set S2, which includes articles relevant to the study from 2013 to 2023, the initial collection of S1 underwent screening and deduplication processes. The difference between the literature databases S1 (obtained through keyword screening) and S2 (obtained based on research relevance) lies in their selection criteria. S1 may include sources with specified keywords that are not directly relevant to the study's objectives; the keywords used are 'patient comfort' or 'comfort experience' or 'factors' or 'influencing' or 'patient satisfaction' or 'healthcare environment' or 'physical environment' or 'social environment' or 'care delivery' or 'nursing care' or 'communication' or 'privacy’ or 'diet' or 'nursing interventions' or 'pain management' or 'patient-centered care’ or ‘well-being' or 'noise' or 'lighting' or 'temperature' or 'emotional support' or 'cultural factors. These keywords can help in conducting a comprehensive literature search related to the factors influencing patient comfort. The S2 dataset, which spans the years May 2013 and May 2023, was used in this study's content analysis of the literature. The selection criteria for S2 were specific and targeted, giving priority to relevance to the research topic and excluding literature that did not align with the research topic, appeared multiple times in multiple databases, originated from non-authoritative sources, or lacked peer review.

Moreover, the process of CiteSpace software analysis involves several steps. Firstly, the S3 dataset, which represents the core literature set from WoS (2013.01–2023.05), is selected. In order to ensure thorough information retrieval, the pertinent literature is subsequently downloaded into a text file with the 'Record information' option setting to 'full record with cited references'. Subsequently, the exported text file is imported into the CiteSpace software. Once imported, the literature data undergoes a cleaning and preprocessing stage. This involves eliminating duplicate entries and correcting any incomplete or inaccurate information to ensure the data's accuracy and completeness. Furthermore, a specific time frame, typically the past decade, is defined to focus the analysis. By setting thresholds, co-occurrence relationships between different keywords are explored, and significant nodes and patterns within the keyword network are identified. Various analysis methods and visualizations, such as time series diagrams and cluster analysis, are selected based on the research objectives. These techniques aid in exploring and analyzing the themes, development trends, and correlations within the literature data. The analysis aims to identify key themes, research hotspots, author collaborations, and to provide valuable insights into the research field. By following this process, the study seeks to gain a comprehensive understanding of the literature, uncover emerging trends, and contribute to the existing knowledge in the field.

The study examined 913 articles on patient comfort, encompassing relevant papers published from January 1977 to May 2023. After conducting a thorough review, 126 of the articles were identified as review papers (with 42 of them being open access), while 790 articles were categorized as research papers (319 of them being open access). To further analyze the literature, the study categorized the findings into indoor physical and social environment dimensions. In the physical environment, factors like air quality, temperature, humidity, lighting, and noise significantly affect comfort. Adequate conditions promote relaxation and rest. In the social environment, care, communication, privacy, and safety impact patients' comfort. Emotional support, interpersonal communication, and a secure environment alleviate discomfort. Care and diet also influence comfort, with warm care and healthy dietary planning reducing discomfort and promoting recovery. Cultural background and personal values shape comfort preferences. Medical staff should respect cultural differences and provide personalized care to enhance patients' overall comfort and well-being.

## Physical environment factors

### Indoor air pollutants

#### Particulate matter

The hospital environment's ambient air quality may vary due to various factors, such as the layout of the hospital's indoor environment, the specific medical treatment received by patients, and the type of ventilation equipment used by the hospital [20]. The air quality in hospital wards may be adversely affected by Numerous typical pollutants. The use of gases, the handling of equipment, and the cutting of tissue during surgical operations can produce particulate matter (PM). Moreover, patients' risk of infection may be impacted by surgical personnel's production of bioaerosols and improper positioning of components in ventilation systems [21, 22]. It is necessary to note that bacteria or viruses attached to the PM surface could deteriorate the air quality, subsequently causing the PM to become infectious. For example, methicillin-resistant Staphylococcus aureus (MRSA) [23], pseudomonas [24], are the bacteria among them. The content of carbon dioxide (CO_2_) and PM_10_ in hospital ICUs have been found to be directly correlated in studies. The levels of coarse particles are associated with both PM_10_ and Fine particle concentrations [25]. By closely monitoring indoor CO_2_ levels and implementing appropriate control measures to minimize CO_2_ concentrations, effective mitigation of the potential danger of airborne infection transmission is possible [26]. Single-use plastics (SUPs) exist in the pharmaceutical and medical fields. Micro-nanoplastics (MNPs) can be released into the environment from medical plastics such as bags, containers, and management equipment, posing potential hazards to patient health [27–29]. Given the numerous technical and analytical challenges involved in extracting, characterizing, and quantifying the concentration of microplastics from environmental samples, there is a possibility of encountering inaccuracies in estimating their levels [27].

In addition, The average indoor PM_2.5_ concentration for a 24-h period shall not exceed more than 25 g/m^3^, according to the World Health Organisation (WHO), and the yearly mean concentration needs to not be higher than 10 g/m^3^. Fine particles, including those in smoke, exhaust, and air pollution, are sufficient in size to be taken readily into the lungs. Elevated levels of PM_2.5_ in indoor air can lead to discomfort, particularly for individuals suffering from COPD and asthma. The presence of airborne PM can significantly affect the respiratory well-being of patients. The incidence and death rate of heart illnesses are both increased when people are exposed to PM [30]. Patients who experienced asthma or allergic respiratory symptoms were more affected by this connection [31]. Particles in the air, such as dust, pollen, or other allergens, can irritate the respiratory tract, causing discomfort, coughing, or difficulty breathing. Lee et al. discovered that the collective concentration of CO_2_, volatile organic compounds (VOCs), and PM_2.5_ in otolaryngology clinics was notably higher compared to the air in orthopedic clinics or reception areas [32]. Because various chemical cleaners and disinfectants are frequently used to sterilise and clean endoscopic equipment, otolaryngologists and patients in otolaryngology clinics have a greater chance of being exposed to indoor air pollution [32]. Reducing the level of PM_2.5_ is a feasible method to reduce the incidence rate, slow down the progress of CORD and reduce the related medical care burden [33]. Ensuring the safety and comfort of patients and staff within medical institutions is of utmost importance, and a crucial aspect of this involves monitoring IAQ and particle concentration [34]. If the particle concentration exceeds the recommended level, it is vital to take measures compliant with standards to improve air filtration or ventilation systems, improving IAQ levels [35].

#### Chemical gases

The research discovered a correlation between using quaternary ammonium compounds (QACs) and an elevated risk of occupational diseases, including asthma and COPD. The overuse of disinfectants such as alcohol, hydrogen peroxide, or bleach showed a link to respiratory tract damage and an increased risk of developing and treating asthma [36]. In hospital hygiene and cleanliness, Leistner et al. found that using probiotics to clean hospital surfaces is better than using other toxic cleaning products to prevent hospital patient infections [37]. By inviting 423 patients from 23 hospitals in Poland to fill out a questionnaire survey and found that 64.8% of patients believed that the hospital ward environment was comfortable, 26.9% felt slightly uncomfortable, and 6.9% felt uncomfortable in the ward. For the odor in hospital wards, 18.8% of patients perceive the air as slightly odorous, and 7.9% perceive it as having a foul odor [38]. In terms of air odor, research has found that one of the most common complaints from patients about hospitals is unpleasant odors, such as mold or dirt, which can cause headaches, and irritation to the eyes, nose, and facial skin, making patients feel uncomfortable and affecting the overall environment of the room [39].

#### Microorganism

Biological contamination in hospital wards encompasses a range of microorganisms, such as viruses, bacteria, fungi, and other pathogens, which can exist in the air. Typical instances of these microorganisms encompass Staphylococcus aureus, including Methicillin-resistant Staphylococcus aureus (MRSA), various species of Pseudomonas, and assorted fungi [40]. The risk of infection significantly increases in specific hospital wards where these microorganisms are prevalent, particularly in hematology/oncology wards, orthopedic wards, surgical wards, neonatal intensive care units (NICUs), and other intensive care units [41]. Microorganisms can cause hospital infections [42], which can survive in patients, visitors, or healthcare professionals and pose a high infection risk to susceptible patients [43–45]. In addition, medical equipment, cleaning agents, and other sources may release chemical pollutants such as airborne pathogens or other toxic chemicals. To mitigate the risk of microbial infections in patients, Ho et al. used skin scale particles to imitate air contaminants. The study revealed that directed airflow systems could minimize people's exposure to contaminants [22]. Gram-positive bacteria like Bacillus cereus, Micrococcus luteus, S. aureus, S. capitis, S. epidermidis, S. haemolyticus, and S. hominis, as well as gram-negative bacteria like A. baumannii, Flavobacterium meningosepticum, and P. aeruginosa, are the main bacteria found in the ward [25]. The research discovered that Gram-negative bacilli and Gram-positive cocci are the most commonly identified bacteria in hospital wards, with a greater frequency of Gram-negative bacilli in ICU wards (75%), based on the investigations of Hemaati et al. [46]. The research done by Mirjalili et al. shows that nanoparticles may adsorb a variety of microbial species. The performance of adsorption can be especially improved by the mixture of CaSO_4_ and CaCO_3_ nanoparticles [47]. Hospitals ensure good IAQ by selecting appropriate ventilation systems, air filtration equipment, and regular maintenance [48]. By referring to research findings on the spread of SARS, hospitals can disinfect the air by increasing outdoor air supply, filtering, disinfector purification, and ultraviolet bactericidal radiation (UVGI), which is crucial for controlling severe acute respiratory syndrome in air conditioning ducts [49]. The studies of Le et al. show that photocatalytic air purification equipment can kill over 99% of bacteria and fungi when air passes through. When placed in an intensive care unit (volume 125 m^3^), the equipment can kill 69% of bacteria and 63% of fungi within 6 h [50]. Besides, using air filters can help remove PM from the air. Compared to other air filters, filters treated with antibacterial preservatives in hospitals have better effects in reducing or delaying mold accumulation [51]. Improving IAQ in hospital wards can be achieved through source control, ventilation, and filtration strategies [1]. Reducing or eliminating indoor air pollution sources can help improve air quality in terms of source control [52]. Mold colonization in high-efficiency air filters and 90–95% of filters is most common on the load surface of equipment, which is also a point source of indoor mold [51]. In brief, consistent monitoring and maintenance of ventilation and filtration systems are necessary for indoor environmental monitoring and upkeep to maintain proper operation and efficiently reduce indoor air pollution.

### Air temperature and relative humidity

Researchers have explored the impact of IAQ in hospitals on respiratory comforts, such as humidity levels and temperature in respiratory comfort. In addition, odor and noise levels can also affect respiratory comfort [39, 53, 54]. Therefore, overall consideration of multiple factors is needed to optimize the patient's respiratory comfort experience in the medical environment. These measures may include air filtration, regular cleaning, HVAC system maintenance, and using low-emission materials and cleaning products. Other factors that promote the patient's comfortable experience include temperature, humidity, and airflow. Extreme temperatures, whether excessively high or low, can result in a negative thermal comfort experience for patients. Insufficient humidity can result in dryness and respiratory system irritation, whereas excessive moisture can promote mold growth and exacerbate respiratory issues. Choi et al. research found that improper stratification of indoor air temperature can affect airflow. For example, when the radiation plate in a displacement ventilation system is heated, it will generate more severe plumes than the human body, locking in the polluted air before reaching the ceiling, thereby controlling the airflow pollutants [55]. It is crucial to have an appropriate ventilation system to ensure proper equipment maintenance and regularly clean to minimize the risk of infection in hospitals to achieve good air quality [56]. In terms of comfort experience, Lawrence et al. believe that patients should experience an equal level of thermal comfort and human comfort. Adjusting temperature and relative humidity, as well as reducing PM, can improve human comfort [57]. Currently, the prediction techniques for assessing patient comfort tend to overlook the influence of patient adaptation. However, through a combination of sensor measurements of indoor environmental quality (IEQ) indicators such as sound, light, and temperature, combined with a survey of 238 patients in two hospital wards, research has uncovered that emphasizing patient adaptation to indoor conditions can effectively minimize variations [58]. The comfort level of patients can be greatly improved by altering the ambient illumination in hospital rooms [59, 60]. The level of air composition that can make patients feel comfortable may vary depending on personal preferences, but generally, the following factors help create a more comfortable indoor air environment. The PMV method proposed by Guo et al. differs from the ASHRAE method for evaluating PMV due to the inclusion of additional factors. Along with taking into account elements like air temperature, mean radiative temperature, air velocity, relative humidity, metabolic rate, and clothing insulation, Guo et al.'s method also takes into account air quality parameters, such as CO_2_ concentration and formaldehyde concentration, enhancing IAQ, boosting freshness of the air, and regulating interior temperature and humidity [61]. Through their experiment, Fang et al. found that the strength of the air odour was only marginally affected by temperature and humidity. However, temperature and humidity significantly influence the impression of IAQ. Strangely, while pollution levels are stable, perception of air quality appears to decline when air temperature and humidity increase [62]. Furthermore, in environmental conditions where humidity exceeds 60% and temperature surpasses 27℃, research conducted by Onmek et al. revealed an inverse relationship between temperature and fungal concentration, with higher temperatures correlating to lower fungal concentrations. Conversely, higher humidity levels were found to be associated with increased levels of fungi [63]. Humidity is also an important influencing factor, and the moisture content in the air can affect the air quality of hospitals. Excessive humidity would promotes the growth of microorganisms and causes problems with equipment and materials. The findings from Ashuro et al.'s study indicate that keeping the relative humidity of indoor air below 50% can mitigate the risk of airborne infections. If the relative humidity is ≤ 40%, the allergens of dust mites in indoor air will decrease, but dry skin, eye irritation, and static electricity will increase with the decrease in humidity, affecting people's comfort [64]. < ASHRAE Standard 170: Ventilation of Health Care Facilities > and < Facility Guidelines Institute (FGI): Guidelines for Design and Construction of Hospitals and Outpatient Facilities > provide different temperature recommendations for the Operating Room, Isolation Ward, Patient Room, and Intensive Care Unit (ICU). According to < ASHRAE Standard 170 > , the temperature range is 20–24 °C (68–75°F), while < Facility Guidelines Institute (FGI) > suggests a range of 20–23.3 °C (68–74°F). However, both guidelines agree on the relative humidity range, which is 30–60%. Regarding temperature regulation, while temperature is a crucial element in determining patients' thermal comfort experience, it is not the sole or predominant one. Humidity, air speed, radiation temperature, garment insulation, and activity levels are other variables impacting thermal comfort [65]. In summary, when researching suitable temperature and humidity ranges for hospitals, it's important to note that these ranges can vary depending on the climate conditions of different countries. Generally, a common guideline for maintaining a comfortable environment in hospitals is a temperature range of 20–24 °C and a relative humidity range of 30–60%.

### Air flow

Maintaining the airflow at a sufficient level can prevent air retention and promote air circulation in the airflow. Tan et al.'s research demonstrated that air curtains were ineffective in reducing particle deposition. The air supply diffuser's surface area was improved from 4.32 to 7.74 m^2^, which led to a 33.3% reduction in particle count. This measure can help prevent surgical site infections [66]. Although the level of indoor air velocity that can make patients feel comfortable may vary depending on the patient's adaptability, overall, the ASHRAE recommends that the air velocity in occupied spaces be less than 0.15 m/s to prevent discomfort caused by airflow. According to studies, when using mobile air supply (MAS), the particle concentration above the patient region gradually decreases from 40 particles/m^3^ to about 0 particles/m^3^, with air velocities ranging from 0.1 to 0.5 m/s. An important finding is that providing air at a speed of 0.5 m/s produces the best results in lowering the concentration of infectious particles above the patient region [67]. In addition, the lateral movement of medical staff affects the airflow and particle distribution in the ward. Tan et al.'s study revealed that walking at a pace of 1 m/s can lower the incidence of hospital infection in burn patients. Concerning the air exchange rate, Liang et al. conducted a study and found that increased airflow in surgical suites leads to significant decreases in CO2, total VOCs, and airborne bacteria levels. Based on their findings, Liang et al. recommend a minimum air exchange rate of 20 ACH for trauma operating rooms and 25 ACH for colorectal operating rooms [68]. According to a study by Zhang et al., while ventilation rate is vital in determining the pollutant levels in the operating room and surgical microenvironment, it is not directly correlated to the decrease in pollutant concentration there. The commonly suggested 20 ACH for maintaining air quality in mixed ventilation operating room surgical microenvironments may require enhancements [69]. The proposed ventilation rates aim to enhance air quality and minimize the presence of airborne pollutants in these surgical settings. Alsved et al. conducted a comparison between two commonly used ventilation techniques, vertical laminar airflow (LAF) and turbulent mixed airflow (TMA), as well as the recently developed temperature-controlled airflow (TcAF) ventilation technology. The findings revealed that TcAF and LAF were more effective than TMA in reducing airborne bacteria, especially near wounds and instrument tables. The unique TcAF ventilation system, similar to LAF, consistently maintains a low level of colony-forming bacteria per unit volume in the air and establishes an improved working environment for healthcare personnel [70]. According to Moreno et al., it is advisable to implement unidirectional airflow in the surgical area to ensure the presence of clean air near the patient and minimize the occurrence of dust, particulate matter (PM), and other pollutants that can cause respiratory discomfort for healthcare workers and patients. The optimal flow rate should ideally fall within the range of 0.25–0.40 m/s for achieving an ultra-clean air environment, considering the importance of cleanliness [71]. According to Choi et al., increasing the downward convective airflow could substantially raise the number of pollutants in inhabited spaces, especially in the lowest section of the room [72].

To put it briefly, sufficient airflow is crucial in facilitating proper air circulation, reducing the likelihood of respiratory problems, and ensuring cleaner air throughout the room [16]. In the indoor environment, airflow control mechanisms such as overcrowding, high temperature, insufficient ventilation, improper waste management, and lack of resources would cause excessive bacteria to stay in the confined space. By controlling the number of patients, caregivers, and visitors, the vital thing is that patients can enjoy a more comfortable and healthy indoor environment with lower microbial levels [45].

### Ventilation system and equipment

Tan et al. conducted a study that demonstrated the efficiency of installing an ensemble of ceiling air diffusers and air curtain ejectors in minimising the deposition of PM on patients (14%), as well as the settling of pollutants in the area where people breathe of medical staff (57%). In addition, installing exhaust grilles helps to remove PM from the ward [73]. Research has found that ceiling exhaust could more effectively remove fine particles than floor exhaust. Improving ventilation design can effectively remove small and large breathing particles in hospital isolation wards. Installing mechanical exhaust fans can transform existing wards using NV into temporary isolation rooms [74]. This may be achieved by carefully analysing how different ventilation patterns affect the control of viral diffusion, the regulation of air quality, and the regulation of temperature in thermal comfort settings [75]. Furthermore, it is crucial to evaluate the applicability of conventional thermal comfort strategies for patients of traditional Chinese medicine in air-conditioned settings with hot temperatures [76]. Lastly, creating a compassionate and supportive environment is also vital in prioritizing patient comfort in hospitals. In terms of ventilation, for obese patients undergoing BPPV treatment, comfortable ventilation can help promote relaxation and sleep [77]. In addition, unpleasant odors can make patients feel uncomfortable, anxious, or embarrassed, harm their overall comfort experience, and may hinder them from seeking care [72, 78]. Therefore, maintaining ideal IAQ in hospitals is crucial for creating a relaxing and healing environment for patients while reducing the possibility of problems and infections [79]. Loud or continuous noise can bring stress and affect their patients' rest and recovery in terms of noise level. When employing ventilation equipment, the incorporation of high-efficiency particulate air filters (HEPA) proves effective in diminishing particle concentration levels, leading to a notable variation in the ACH within the ward. However, when using the filter maximum airflow, the noise level in the room increases [80]. Maintaining low noise levels and minimizing interference can improve the patient's comfort and perfect their sleep experience [54, 81, 82].

There are several ways to innovate the management methods of indoor air environments, which can provide patients with a better comfortable experience. (1) Using air purification equipment: Common air pollutants in wards, such as CO_2_, can harm IAQ and affect patient comfort and health [7]. By utilizing modern air purification equipment that effectively eliminates bacteria, fungus, and viruses by destroying their DNA and RNA, hospitals can significantly reduce the length of a patient's stay. This not only leads to cost savings but also improves IAQ, resulting in a decreased incidence of hospital-acquired infection (HAI) and a reduced risk of infection and respiratory discomfort [83]. Controlling humidity levels helps to prevent large particles from entering the indoor environment. Qi and Deng have successfully designed multi-input multi-output (MIMO) controllers that demonstrate remarkable proficiency in regulating indoor air temperature and humidity. These controls effectively adapt the compressor speed and supply fan speed of the DX A/C system in response to variations in temperature and humidity to guarantee precise control over IAQ [84]. When the concentration of indoor microbial pollution is high, air filtration and combining ultraviolet sterilization to better control air quality [85]. (2) Using CO_2_ sensors: CO_2_ concentration serves as an alternative indicator to measure the risk of respiratory pathogen transmission during morning care and stay, and improving air exchange rates can also help reduce indoor CO_2_ concentration in hospital rooms and improve IAQ. Installing a CO_2_ sensor can monitor the level of CO_2_ and provide ventilation when the level exceeds a certain threshold, preventing excessive energy consumption caused by continuous ventilation [86]. (3) Making use of personalised comfort systems: Modify the system to accommodate each patient's unique demands (such as temperature, humidity, and airflow). (4) Using real-time monitoring systems: The system can track IAQ. For example, a low concentration of pollution sources near the hospital bed may be maintained by vertically mounting the sensing device of the all-air wall induction unit device in the four corners of the ward [87]. In addition, evaluating and managing thermal comfort and human comfort under NV, passive split ventilation, and active ventilation systems are beneficial for the health of patients and residents [57]. Overall, air purification equipment, CO_2_ sensors, personalized comfort systems, and real-time monitoring systems can control indoor PM levels, enabling patients to enjoy a more comfortable and healthy indoor environment. Table 1 shows the environmental control systems related to respiratory and thermal comfort of patients.

**Table 1:** Summary of several kinds of environmental control systems

Type	Description	References
ACMV systems	Better IAQ control performance over chemical and particulate pollutants	[88]
	The presence of a table between two users resulted in an approximate 50% increase in the user's exposure risk (DV system) and a 22% increase (MV system)	[89]
	Complex MV system could reduces the chance of hospital-acquired infection (HAI) airborne transfer	[90]
	NV and MV systems could provide fresh air and remove polluted air from the indoor environment	[91, 92]
Intelligent environmental monitoring system	Send messages to distant users and operate ventilation and air purifiers with effectiveness using mobile devices	[93]
	Independent temperature and humidity control, individualised fresh air ventilation, and increased indoor comfort	[94]
The monitoring system	Utilizing WSN with web interface to effectively monitor air quality of environmental parameters, providing patients with a more comfortable environment	[95]
	Monitor the concentration of indoor and outdoor air pollutants to limit the sources of indoor PM and other pollutants	[96]

After comparing the environmental monitoring systems presented in Table 1, it becomes evident that the ACMV system plays a crucial role in ensuring good IAQ by effectively controlling chemical and particulate pollutants. On the other hand, the intelligent environmental monitoring system operates ventilation and air purifiers using mobile devices, enabling independent control of temperature and humidity for personalized ventilation and enhanced indoor comfort. Meanwhile, the monitoring system utilizes wireless sensor networks (WSN) and network interfaces to continuously monitor environmental parameters, ensuring a comfortable environment for patients. The primary objective of these systems is to enhance indoor comfort and air quality. While the ACMV system focuses on controlling pollutants, the intelligent environmental monitoring system takes it a step further by enabling operation and personalized ventilation through mobile devices. Additionally, the monitoring system employs WSN to monitor air quality and limit pollutant sources. These systems are supported by relevant references, serving as valuable sources of information for related research.

Based on information provided, it can be inferred that the ventilation system in hospitals is responsible for delivering the best possible thermal comfort and reducing the airborne transmission of illnesses associated with healthcare. Currently, most hospitals employ mechanical ventilation systems, and innovative management approaches for IAQ have emerged. The technology for removing particulate matter from the air is relatively mature. However, there is a lack of ventilation devices capable of effectively eliminating irritating chemicals such as Hypochlorous acid released during indoor cleaning and formaldehyde emitted from furniture and equipment. For respiratory comfort and hospital-acquired infection (HAI) prevention, monitoring the amount of contaminants in indoor air is crucial.

### Social environmental factors

#### Nursing and diet

In terms of nursing, patients may experience pain or discomfort due to their medical condition and treatment procedures, which can prolong hospitalization. Effective pain management can help alleviate this discomfort and improve patients' self-cultivation and sleep experience [54]. In addition, the quality of care patients receive in the hospital can also affect their comfort experience [15, 97]. The study discovered through interviews that were semi-structured in nine critical care nurses that patients who received sympathetic and meticulous care from medical service providers had higher satisfaction with medical staff and higher nursing efficiency, which may shorten their stay in the ICU [98]. Physical healing is aided by compassionate nursing care for burn sufferers [99]. In terms of diet, treatment regimens and dietary restrictions affect patients' lives [100], the patient's diet plays a crucial role in their overall comfort and satisfaction regarding food and nutrition. Ingesting comforting meals with a high caloric content has been found to alleviate discomfort associated with neurohypoglycemia in patients with Addison's disease [101]. Similarly, a study demonstrated that enjoyable eating experiences can reduce pain sensations in patients undergoing targeted tumor therapy [102]. For individuals diagnosed with colorectal cancer, incorporating a dietary plan that includes a higher intake of dietary fiber can effectively alleviate discomfort and expedite the recovery process [103]. Consequently, providing nutritious and appetizing meals tailored to meet specific dietary needs can greatly enhance the patient's comfort and overall experience.

#### Privacy and communication

In terms of privacy, patients may feel vulnerable internally during hospitalization, so ensuring respect for their privacy and confidentiality is crucial. Saleem et al. conducted structured interviews with 571 patients in the emergency room. The study found that 10% of patients would refuse physical examination because of privacy issues, especially in an acute environment with a high incidence rate and mortality which is vital to pay attention to [104]. Closing the curtains and the door during examination or surgery can make patients feel more comfortable [105]. Hartigan et al.'s study discovered that altering the atmosphere can enhance patients' experiences in the emergency room. By replacing the patient area covered by curtains in the emergency room with a walled compartment, the proportion of patients who believed their privacy was adequately protected increased from only 21% (*n* = 16) to 89% (*n* = 73) [106]. Research has found that by expanding the treatment space area of the emergency room, the proportion of patients who can unintentionally hear conversations about themselves or other patients has decreased from 36 to 14%, which protects the privacy and confidentiality of patients to some extent [107].

In the communication aspect, patients can obtain emotional support through consultation services or other communication methods, which can help achieve a surprisingly comfortable experience [108]. Through semi-structured interviews with four focus groups (*N* = 19), The research of Milette et al. study found that receiving care from family, friends, and others can help patients better recover and cope with the disease [109]. Effective communication and obtaining information about the condition and treatment can help patients feel more comfortable and controllable [110]. By analyzing 1241 valid questionnaires from 500 outpatient patients and 800 inpatients regarding their experiences during treatment, the study found that, in addition to hospital differences, the age and occupational status of patients significantly affected their experiences (*p* < 0.05), and there was a strong correlation between respecting patient preferences and patient satisfaction with the treatment experience [111]. By utilizing patient feedback and meeting their preferences as environment management strategies, hospitals can create a comfortable experience for patients [112]. In short, substantial communication of patient feedback between healthcare professionals and patients can help create a comfortable and healthy environment and enhance patient comfort.

## Results

### IAQ standards

The following are the current standards and guidelines for hospital indoor control quality in Australia, the United States, Canadian, the European Union (EU), China, Japan, Malaysia, and Singapore. Studies have discovered that subjective assessments from patients and staff are far more reliable than objective measures to predict indoor comfort levels [113].

Various countries and authorities have implemented standards and guidelines to address indoor environmental quality, covering aspects such as pollutants, ventilation, temperature and humidity control, and health management [114]. The objective is to provide the residents of various buildings, especially medical institutions, with a secure, healthy, and comfortable interior environment.

### Analysis of countries and cooperative relationships

**Fig. 2:**
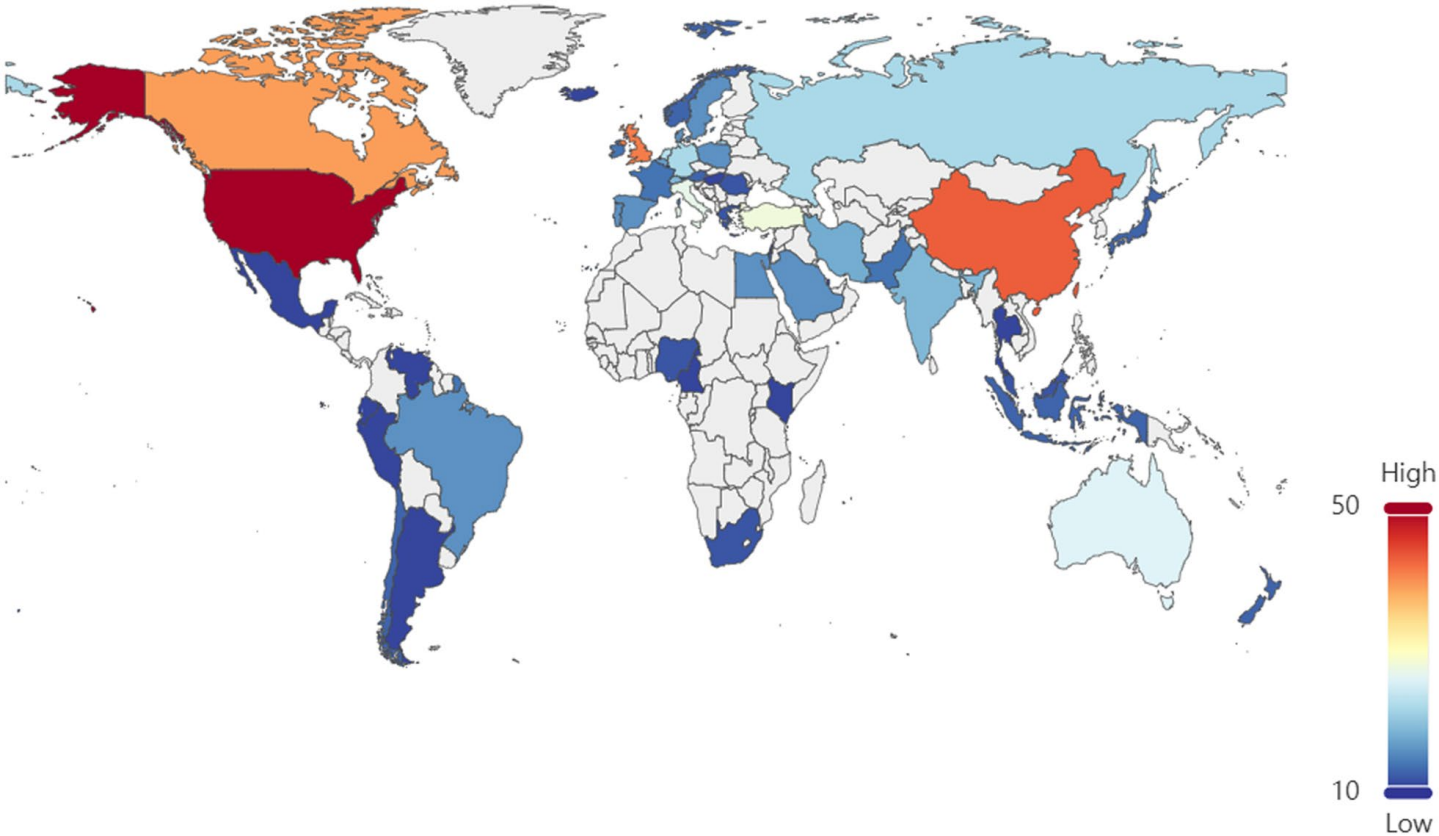
Distribution map of published articles on factors affecting patient comfort experience in different countries over the past decade (The numerical unit is the number of articles)

**Fig. 3:**
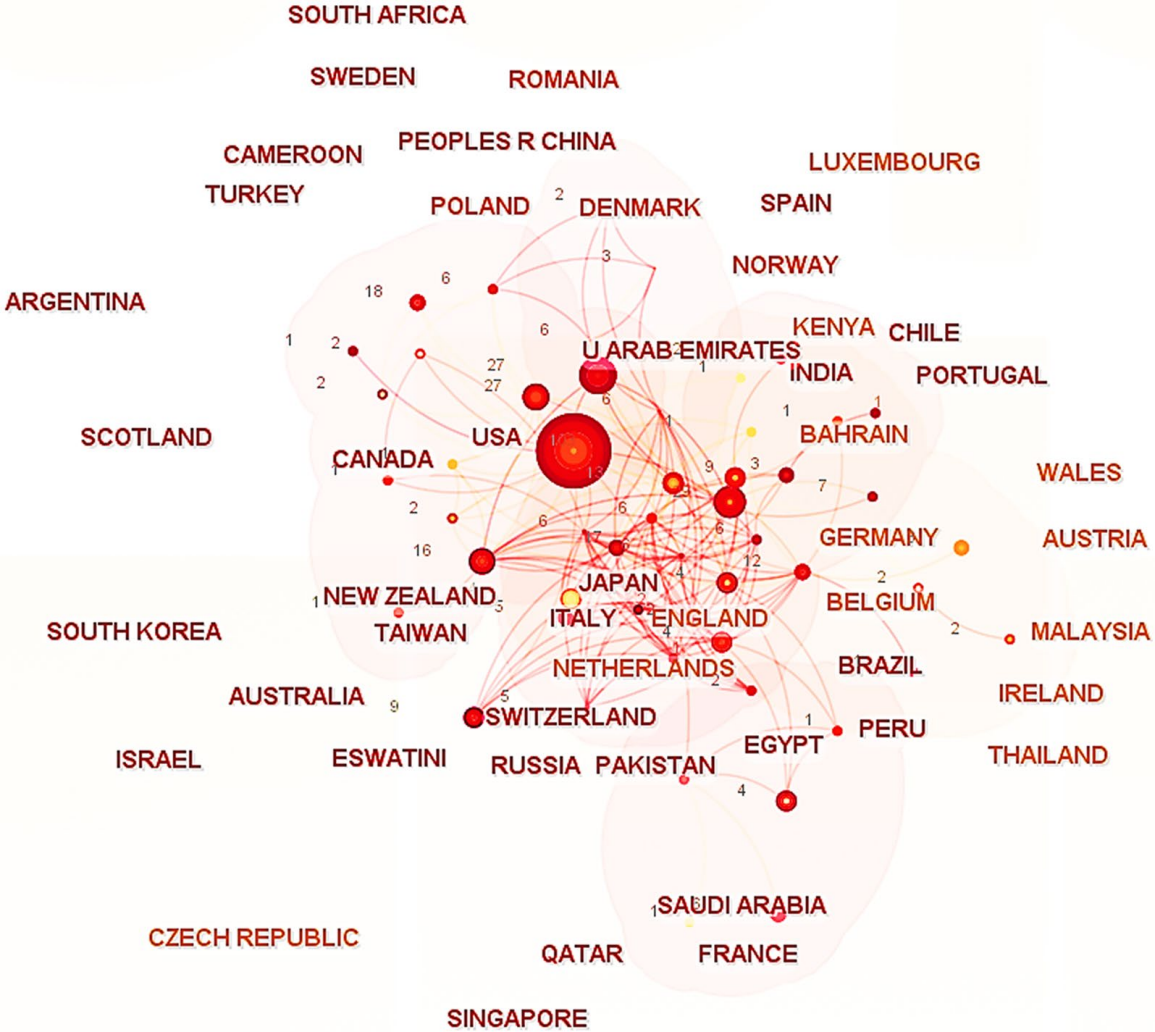
Collaborative relationships between researchers from major countries

By examining the total number of publications in the S1 database (425 papers), Fig. 2 prominently displays the distribution of literature on factors affecting patient comfort experience in various countries over the past decade. It is evident that American researchers have made the most significant contributions to research on patient comfort. Following closely are researchers from China, the United Kingdom, and Canada, demonstrating their significant participation and contributions in this field. Figure 3 illustrates the collaborative relationships between researchers from major countries. It is clear that the United States has established strong partnerships with European countries and actively participates in collaborative research projects. The United States, the United Kingdom, and Japan serve as key driving forces and central hubs for knowledge exchange in the field of patient comfort research.

### Analysis of influencing factors

**Fig. 4:**
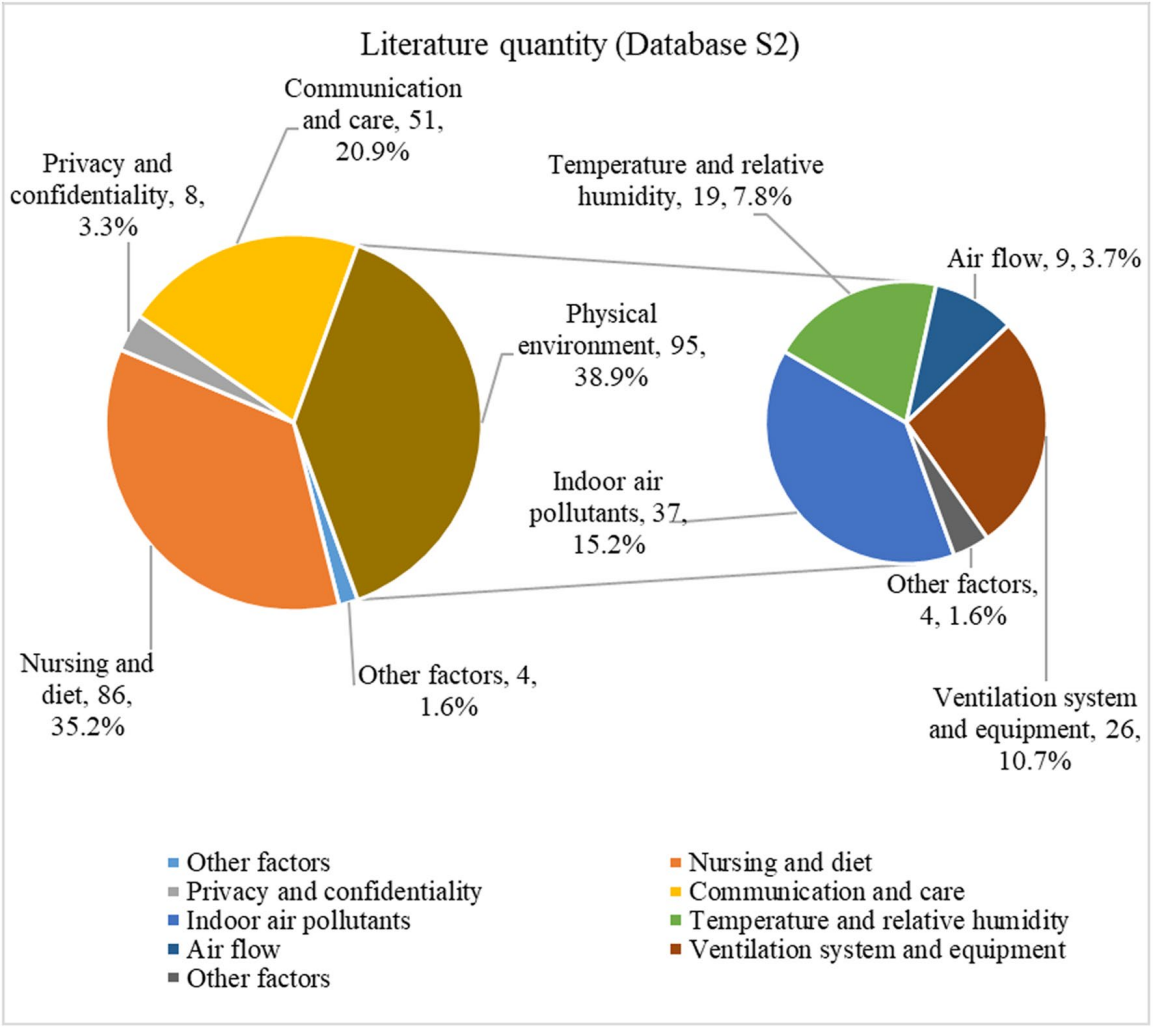
Conduct a research category analysis of the articles relevant to the study from 2013 to 2023 in database S2

Through the organization and analysis of 244 articles from the literature database S2, it has been found that factors influencing patient comfort experience primarily fall into several categories: indoor physical environment, nursing and nutrition, privacy, and communication. The interior physical environment elements contain a wide range of sub-factors, such as indoor air pollutants, temperature, relative humidity, airflow, ventilation systems. Considering Fig. 4, the distribution of article categories is represented as a percentage of the total. Most researchers contend that the physical environment (38.9%) is closely tied to patients' comfort, even though there is relatively less literature available on air pollutants in comparison to nursing and diet (35.2%), communication and care (20.9%), which only account for a share of 15.2%.

**Fig. 5:**
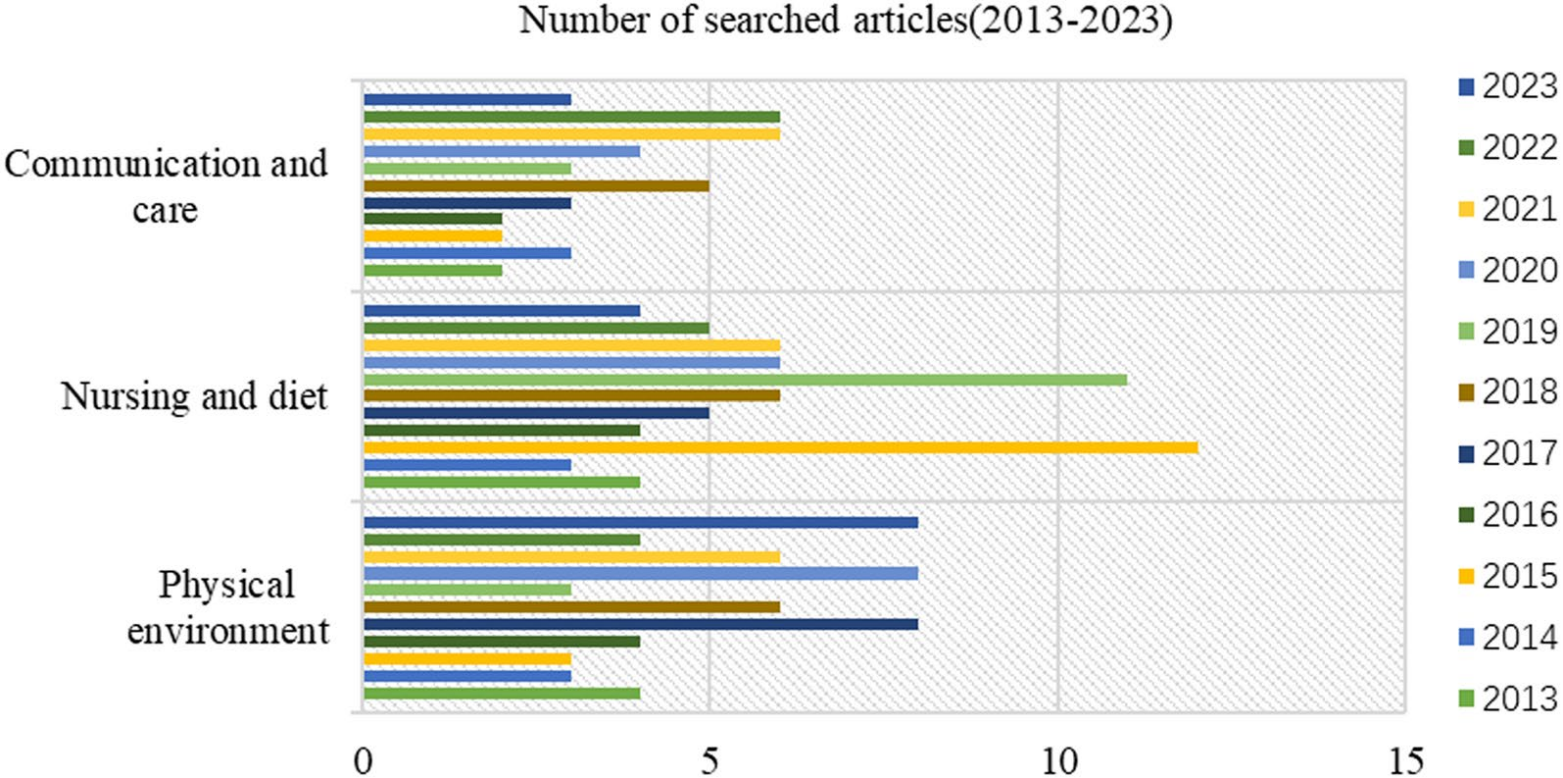
Analysis of literature quantity on the main influencing factor categories

Figure 5 analyzes the main factors influencing patient comfort experience based on the S2 database over the past decade, including the indoor physical environment, nursing and dietary factors, and communication and care. The analysis reveals a substantial increase in publications focused on the indoor physical environment, indicating its recognition as an emerging and critical research area. Nursing and dietary factors exhibit noticeable fluctuations, reaching peak publication volumes in 2015 and 2019. The literature on communication and care influences shows more moderate changes but still demonstrates a positive trend over time.

### Keywords and cluster analysis

**Fig. 6:**
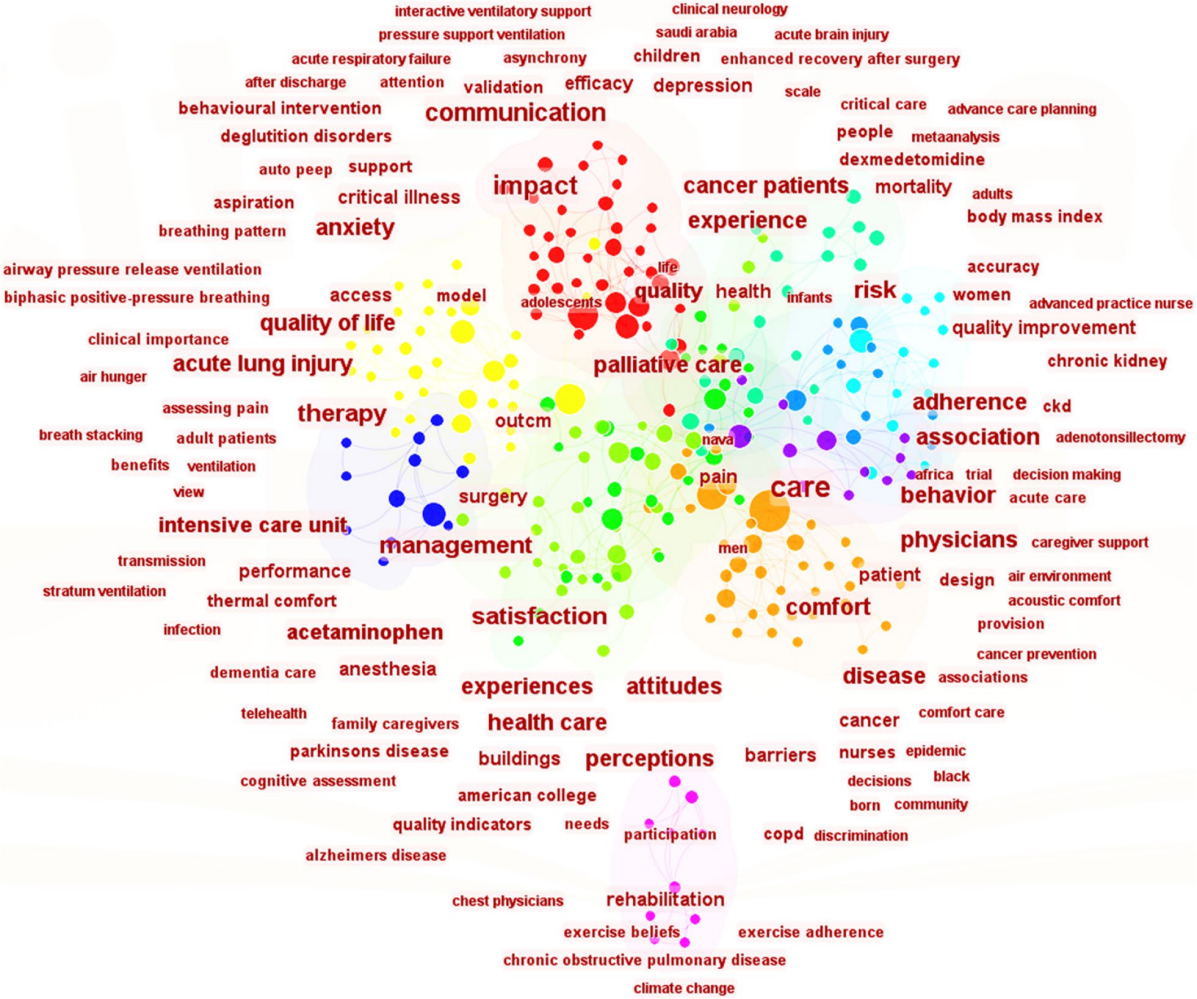
Analysis of keyword frequency in literature

**Fig. 7:**
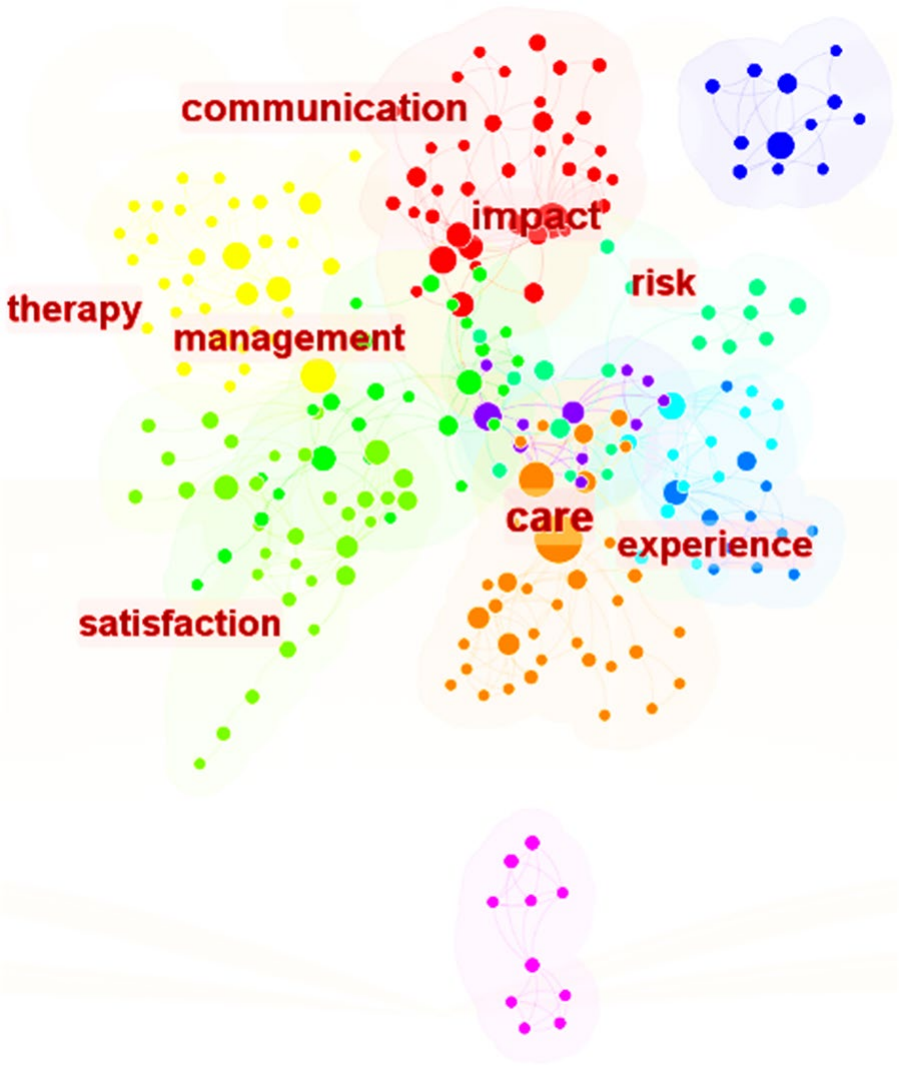
The top eight keywords with the highest frequency of occurrence

**Fig. 8:**
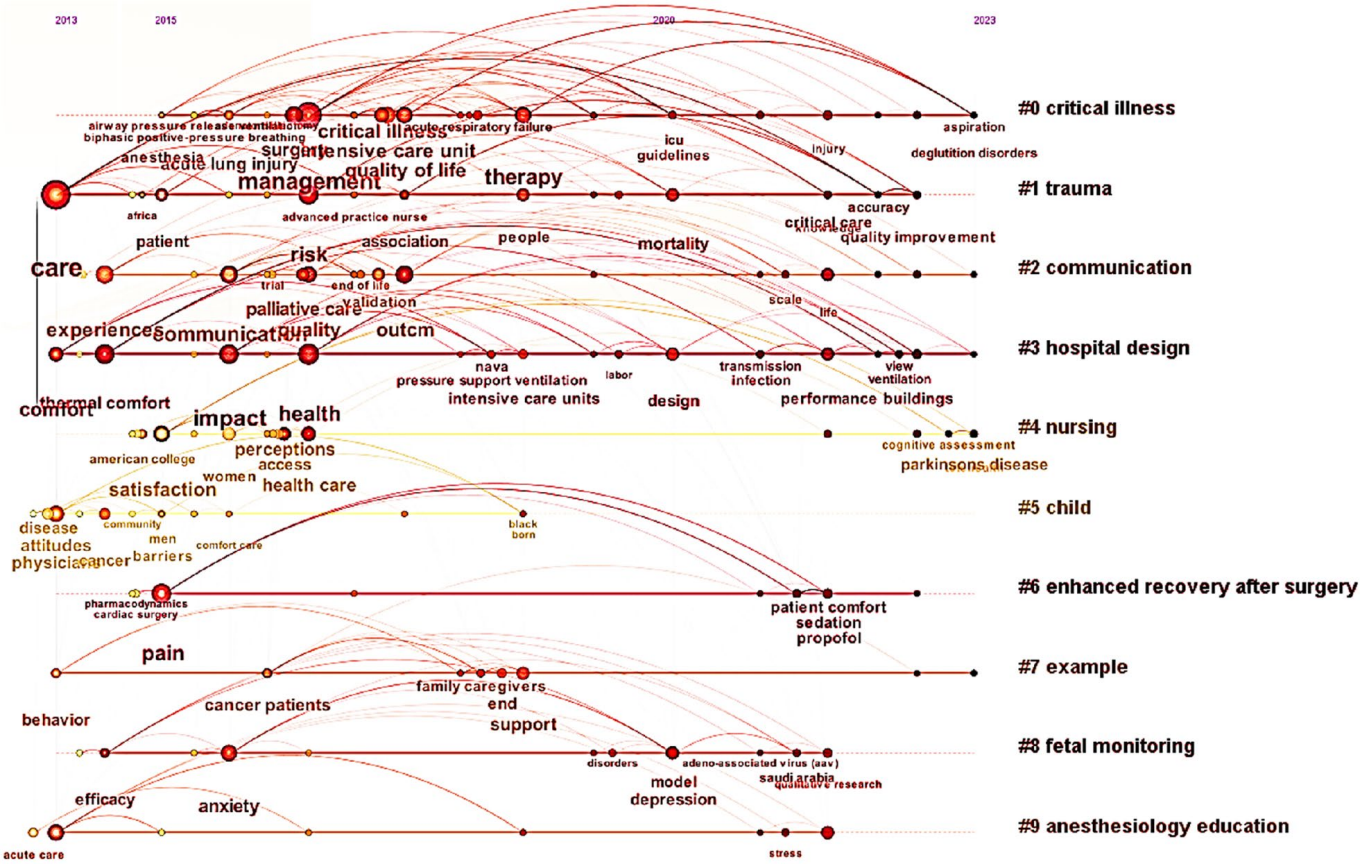
Research Trends over Time

The examination of the literature in the S3 database yielded the findings presented in Figs. 6, 7, 8. Figure 6 shows the analysis of keyword frequencies, highlighting commonly mentioned keywords. The size of the nodes corresponds to keyword frequency, with larger sizes indicating higher occurrence rates. Figure 7 displays the top eight keywords with the highest frequency of occurrence. The keywords include communication, management, risk, experience, satisfaction, impact, therapy, and care. Figure 8 displays the clustering analysis of thematic literature over the past decade. The numbers marked with "#" represent literary themes, with smaller numbers indicating more prevalent themes. The top five categories with higher research themes are critical illness, trauma, communication, hospital design, and nursing, commonly found in literature on patient comfort experiences. The vocabulary along the horizontal line indicates the order of keyword appearance, with larger font sizes indicating more frequent mentions. By observing nodes with larger font sizes in the CiteSpace timeline graph, it becomes evident that the keywords in the literature have undergone significant changes between 2013 and 2023. Initially, the prominent theme words included 'care', 'thermal comfort', 'communication', 'health', and 'pain'. However, over time, these themes have been consistently supplemented with new keywords, reflecting the evolving research landscape. For instance, the inclusion of keywords such as 'mortality rate', 'design' (environment), and performance' (ventilation) indicates the expansion of research interests and the exploration of new topics. These additions demonstrate a growing focus on factors like mortality rates, environmental design, and performance aspects of ventilation systems.

To summarize, the analysis of literature database S3 provides valuable insights into the key factors influencing patient comfort. It encompasses both enduring themes and emerging trends in the field. By thoroughly reviewing and analyzing multiple relevant studies and datasets, this study has established that air quality and indoor environmental quality are crucial determinants of the patient comfort experience. Moreover, it underscores the significance of patient care, health, effective communication, and compassionate care in fostering a positive and comfortable experience. This study seeks to increase patient comfort by addressing these elements in order to broaden our understanding of it.

## Discussion

From Table 2, it is clear that some countries prioritize IAQ by actively implementing measures for its continuous improvement and protection. The fact that the US leads the world in terms of the number of IAQ management standards and recommendations shows how seriously it takes protecting indoor air quality and human health and upholding high IAQ standards. By combining Figs. 2 and 3, one can infer that the strong cooperative relationships depicted in Fig. 3 are likely contributing to the significant research contributions of American researchers (as shown in Fig. 2). The active participation of researchers from the United Kingdom and Canada in patient comfort research (Fig. 2) may also be influenced by their collaborative relationships with other countries (Fig. 3). This suggests that collaboration plays a vital role in fostering research advancements and facilitating knowledge exchange in the field of patient comfort. According to Fig. 4, considering the potential impact of indoor air pollutants on patients' health recovery, it becomes evident that this relevance cannot be disregarded. This includes the effects of indoor air particles, fungi, and bacteria within hospitals on the risk, mortality, and incidence rates of patients contracting microorganisms. Prioritizing indoor air pollutants is an approach with considerable potential for enhancing patient comfort, especially in terms of patients' health recovery. Furthermore, according to Figs. 6, 7, 8, in the most recent years, the emphasis has shifted towards topics related to ‘quality improvement’, and ‘cognitive assessment’. Comparing Figs. 4 and 8, it can be observed that while the physical environment, nursing, and communication are commonly mentioned, future research can focus more on the comfort experience of trauma patients. For future research, extensive studies conducted by researchers, hospital managers, and operators have significantly enhanced our understanding of IAQ in medical environments. However, these studies have identified common flaws in surveys regarding patients' comfort experiences. To effectively describe the impact of influencing factors on the rate of change in patient comfort, accurate data is essential. Many studies have focused solely on specific pollutants, neglecting important aspects such as ventilation modes and the comprehensive removal efficiency of substances like formaldehyde and micro/nanoplastics. It is crucial to conduct longitudinal studies to capture the temporal changes in patient comfort. To improve patients' thermal and respiratory comfort and reduce the risk of hospital-acquired infections (HAIs), it is crucial to prioritize the improvement of ventilation systems as a vital component of the physical environment. In the social and cultural environment dimension, healthcare providers can enhance the quality of care by emphasizing patient-centered approaches, promoting physical health recovery, and prioritizing patient comfort.

**Table 2:** National/institutional standards and guidelines for IAQ

Country/authority	Standards and guidelines
Australia National Health and Medical Research Council (NHMRC)	The Indoor Environmental Quality Standards and Guidelines
United States Environmental Protection Agency (EPA)	The IAQ Guidelines for Indoor Buildings
	The Indoor Air Pollution Reference Handbook
American Society of Heating, Refrigerating, and Air-Conditioning Engineers (ASHRAE)	ASHRAE 170: Ventilation of Health Care Facilities
United States Facility Guidelines Institute (FGI)	Guidelines for Design and Construction of Hospitals and Outpatient Facilities
Canadian Council of Ministers of the Environment (CCME)	The IAQ Guidelines
	Indoor Environmental Quality Standards
	Indoor Mold and Fungal Growth Health Guidelines: Interpretation and Application
European Union	The IAQ Directive (2008/50/EC)
	Indoor Environment and Health Directive (2002/44/EC)
China	Guobiao Standards-GB/T 18883–2002 IAQ Standard
	Huanjing (Environmental) Standards-HJ 581–2010 Hygienic Standard for Indoor Environment of Hospitals
	Guobiao Standards-GB/T 18801–2015 Indoor Environmental Pollution Control Standard
Japan	The Indoor Environmental Health Standard
	Japanese Industrial Standards (JIS) Z 8901–2019 Method for the assessment of IAQ
Ministry of Health Malaysia	Guidelines for Health Facilities and Environmental Hygiene Control Authority
Institute of Architects Malaysia	Guidelines for IAQ in Buildings
Malaysia Department of Environment (DOE)	IAQ Code of Practice 2010 (IAQ ICOP 2010)
Singapore National Environment Agency (NEA)	Code of Practice on IAQ for Air Conditioned Buildings

## Conclusion

Following comprehensive examinations and analyses of various studies and datasets, this research uncovered two dimensions crucial to patient comfort: the indoor physical environment and the social environment. This study underscores the pivotal role that nursing and communication play within the social environment in enhancing patient comfort. The physical environment shares a close relationship with patient comfort, and prioritizing indoor air pollutants in patients' health recovery emerges as a method with substantial potential to enhance patient well-being, even though there may be limited literature in this area. These findings provide valuable guidance for improving medical services and optimizing the overall patient experience. However, the review also revealed common flaws in patient comfort experience surveys, such as the absence of standardized measurement methods for assessing hospital physical and social environments, which impedes the comparison of research results. Accurate data is indispensable for effectively describing the impact of influencing factors on the rate of change in patient comfort. Future studies should prioritize individuals, especially those who have experienced trauma, to enhance their comfort levels through the provision of supportive physical and social environments. This approach will ultimately help alleviate their suffering. To promote research in critical illness, trauma, communication, hospital design, and nursing, fostering enhanced international communication and collaboration among researchers from various nations is essential.

Regarding the study's contribution to knowledge, it highlights the significant potential for indoor air pollution to enhance patients' quality of life while they are receiving therapy. For patients to be well-cared for, there must be effective care and communication. The study suggested a course for future research and emphasised the significance of trauma, communication, hospital design, and nursing. It also suggested focusing on patients with particular nursing requirements. To further examine the determining aspects of patient comfort, this study employs a unique approach that involves doing extensive research across a variety of topics and integrating the information from several academic sectors. This study also explicitly focuses on enhancing the interior environment, particularly addressing the issue of indoor air pollution, which is a relatively new area of research and has the potential to have practical applications as well as improve the medical environment. Finally, this study identifies key topics for more investigation. This forward-thinking approach is new in encouraging the direction of research in adjacent domains. In conclusion, this study offers fresh and insightful perspectives on the multidimensional influencing variables of patient comfort, the enhancement of the interior environment, and future research prospects.

## Data Availability

Not applicable.
